# Another piece of the Zika puzzle: assessing the associated factors to microcephaly in a systematic review and meta-analysis

**DOI:** 10.1186/s12889-020-08946-5

**Published:** 2020-06-01

**Authors:** Luciana Guerra Gallo, Jorge Martinez-Cajas, Henry Maia Peixoto, Ana Carolina Esteves da Silva Pereira, Jillian E. Carter, Sandra McKeown, Bruno Schaub, Camila V. Ventura, Giovanny Vinícius Araújo de França, Léo Pomar, Liana O. Ventura, Vivek R. Nerurkar, Wildo Navegantes de Araújo, Maria P. Velez

**Affiliations:** 1 Postgraduate Program in Tropical Medicine, University of Brasilia, Brasilia, Brazil; 2 Department of Obstetrics and Gynecology and Department of Public Health Sciences, Queen’s University, Kingston, Canada; 3 Department of Medicine, Queen’s University, Kingston, Canada; 4 Program of Evidence for Policies and Health Technologies, Oswaldo Cruz Foundation, Brasilia, Brazil; 5 Bracken Health Sciences Library, Queen’s University, Kingston, Canada; 6 Centre Pluridisciplinaire de Diagnostic Prénatal de le Martinique, Maison de la Femme, de la Mère et de l’Enfant, University Hospital of Martinique, Fort-de-France, Martinique; 7 Department of Scientific Investigation, Altino Ventura Foundation, Recife, Brazil; 8 Brazilian Ministry of Health, Brasilia, Brazil; 9 Materno-fetal and Obstetrics Research Unit, Département “Femme-Mère Enfant”, University Hospital, Lausanne, Switzerland; 10 Department of Obstetrics and Gynaecology, Centre Hospitalier de l’Ouest Guyanais Franck Joly, Saint-Laurent-du-Maroni, French Guiana; 11 Department of Pediatric Ophthalmology and Strabismus, Altino Ventura Foundation, Recife, Brazil; 12 Department of Tropical Medicine, Medical Microbiology and Pharmacology, John A. Burns School of Medicine, University of Hawaii at Manoa, Honolulu, USA

**Keywords:** Zika virus, Microcephaly, Pregnancy, Congenital disease, Risk factors, Systematic review, Congenital Zika syndrome

## Abstract

**Background:**

Although it is known that Zika virus (ZIKV) infection during pregnancy may lead to microcephaly in the fetus, the prognostic factors associated with this tragic disorder remain unclear. We conducted a systematic review and meta-analysis to assess the prognostic factors associated with the incidence of microcephaly in congenital ZIKV infection.

**Methods:**

We conducted a comprehensive search in Ovid MEDLINE, Ovid MEDLINE (R) Epub ahead of print, Embase, Embase Classic, Web of Science, CINAHL, Cochrane CENTRAL, LILACS, and various thesis databases to identify human studies reporting microcephaly associated with congenital ZIKV infection. We requested primary data from the authors of the included studies to calculate summary estimates and conduct the meta-analysis of the most prevalent factors.

**Results:**

We screened 4106 titles and abstracts, and identified 12 studies for inclusion in the systematic review. The assessment of ZIKV infection and the definition of microcephaly varied among studies. A total of 6154 newborns/fetuses were enrolled; of those, 1120 (18.20%) had a diagnostic of ZIKV infection, of which 509 (45.45%) were diagnosed with microcephaly. Nine studies addressed the link between congenital ZIKV infection and neurological findings in newborns/fetuses. Half of the studies provided primary data. Three out of 11 factors of interest seem to be prognostic factors of microcephaly: infant’s sex – males compared to females: Relative Risk (RR) 1.30, 95% Confidence Interval (95% CI) 1.14 to 1.49; the stage of pregnancy when infection occurred – infection in the first trimester of pregnancy compared to infection at other stages of pregnancy: RR 1.41, 95% CI 1.09 to 1.82; and asymptomatic infection compared to symptomatic infection during pregnancy: RR 0.68; 95% CI 0.60 to 0.77.

**Conclusion:**

Our findings support the female-biased resistance hypothesis and reinforce the risk associated with the stage of pregnancy when ZIKV infection occurs. Continued surveillance of ZIKV infection during pregnancy is needed to identify additional factors that could contribute to developing microcephaly in affected fetuses.

**Protocol registration:**

This systematic review was registered with the International Prospective Register of Systematic Reviews (PROSPERO), registration no. CRD 42018088075.

## Background

In 2016, the World Health Organization (WHO), for the fifth time in its history, declared a Public Health Emergency of International Concern due to the recognition of the Zika Virus Congenital Infection [1]. After a “pandemic that surprised the world” [2], several studies reported an association between Zika virus (ZIKV) infection during pregnancy and congenital abnormalities [3–6]. Microcephaly is considered the “tip of the iceberg” in Congenital Zika syndrome (CZS), which defines a more complex spectrum of anomalies related to ZIKV congenital infection [7, 8]. When present, microcephaly indicates a neurogenesis failure that varies in severity [9, 10].

Brazil saw the largest outbreak of ZIKV infection and was the first country to investigate the relationship between ZIKV congenital infection and microcephaly. Between November 2015 and November 2018, almost 17,000 suspected cases of CZS were reported to the Brazilian Ministry of Health. Of those, 2819 were confirmed cases – either tested by laboratory methods or based on clinical-epidemiology evidence [11]. Brazilian data revealed a frequency of microcephaly up to 24 times higher following Zika virus infection during pregnancy (PZIK) [12]. In 2016, a study that reviewed data from the 2013–2015 outbreak of ZIKV in French Polynesia estimated a microcephaly risk ratio of 53.4, caused by ZIKV infection in the first trimester of pregnancy [13]. In the Hawaiian Islands, one of the territories closest to French Polynesia, there was a threefold increase in the microcephaly rate between 2005 (4.8 cases per 10,000 total births in 2005) [14] and the 2007–2013 period (14.7 per 10,000 total births) which coincided with the known outbreaks of ZIKV in the Pacific that started in 2007 [13, 15, 16]. Worldwide, a systematic review estimated a prevalence of microcephaly of 2.3% among all ZIKV infections during pregnancy [17].

Although the association between PZIK and microcephaly is considered a “scientific consensus” [6, 18], variations on the risks within geographical areas and population groups have been observed [17, 19]. It has been discussed that some factors may act as effect modifiers, increasing the risk of neurological damage [20]. However, there is still a lack of evidence on cofactors or component causes that act as associated risks or preventive factors in the development of birth defects [21–23]. To address this gap in knowledge, this systematic review and meta-analysis aim to identify maternal and fetal prognostic factors associated with microcephaly in fetuses and newborns from mothers infected with ZIKV during gestation.

## Methods

### Protocol and registration

The systematic review protocol was registered on February 21, 2018, in the PROSPERO (International Prospective Register of Systematic Reviews) database under the number CRD42018088075 [24]. The Preferred Reporting Items for the Systematic Reviews and Meta-Analysis (PRISMA) [25] checklist was filled out and can be found in the Additional Table 1.

**Table 1:** Characterization of the selected studies

Authors (year)	Type of study	Country	Period of Study	Aim of the study	Definition of Zika virus positive	Quality assessment
Aragão, MFVV et al. (2017) [26]	Case-control	Brazil	Dec 2015 – Nov 2016	*“to review neuroimaging of infants to detect cases without microcephaly and compare them with those with microcephaly”*	Laboratory evidence: ZIKV IgM in cerebral spinal fluid and/or serum samples	Satisfactory
Schaub, B et al. (2017) [27]	Case-control	Martinique	Jan 2016 – Nov 2016	*“to describe the early ultrasound markers and progression of the fetal cerebral insults during the pregnancy”*	Laboratory evidence: ZIKV RNA (RT-PCR) or ZIKV IgM or IgG in serum, amniotic fluid, placenta, amnion, cerebrospinal fluid, or brain samples	Satisfactory
Krow-Lucal, ER et al. (2018) [28]	Case-control	Brazil	Aug 2015 – Feb 2016	*“to assess the association of microcephaly and Zika vírus”*	Laboratory evidence: ZIKV IgM in blood samples. Presumed infection also acceptable.	Satisfactory
Honein, M A et al. (2017) [29]	Cohort	USA	Dec 2015 – Sep 2016	*“to estimate the preliminary proportion of fetuses or infants with birth defects after maternal Zika virus infection by trimester of infection and maternal symptoms”*	Laboratory evidence: ZIKV RNA (rRT-PCR), ZIKV IgM (PNRT ≥10) and either a DenV- IgM or a DenV PRNT< 10 (or both) in serum, placenta or other tissue samples	Good
Kumar, M et al. (2016) [30]	Case-control	USA	2009–2012	*“to find a link between ZIKV infection and babies born with microcephaly” in Hawaii*	Laboratory evidence: ZIKV IgM and IgG in serum samples	Good
Brasil, P et al. (2016) [31]	Cohort	Brazil	Sep 2015 – May 2016	*“to describe clinical manifestations in mothers and repercussions of acute ZIKV infection in infants”*	Laboratory evidence: ZIKV RNA (RT-PCR) in serum and/or urine samples	Good
Pomar, L et al. (2017) [32]	Cohort	French Guiana	Jan 2015 – Jul 2016	*“to establish the incidence of fetal central nervous system (CNS) anomalies (including microcephaly), signs of congenital infection and fetal loss in pregnant women infected with Zika virus (ZIKV) and noninfected pregnant women in western French Guiana”*	Laboratory evidence: ZIKV RNA (RT-PCR) or ZIKV IgM or PRNT in serum, placenta, urine, amniotic fluid and fetal samples	Satisfactory
Sanz Cortes, M et al. (2018) [33]	Cohort	Colombia	Dec 2015 – Jul 2016	*“(1) to assess the prevalence of microcephaly and the frequency of the anomalies that include a detailed description based on ultrasound and magnetic resonance imaging in fetuses and ultrasound, magnetic resonance imaging, and computed tomography imaging postnatally, (2) to provide quantitative measures of fetal and infant brain findings by magnetic resonance imaging with the use of volumetric analyses and diffusion-weighted imaging, and (3) to obtain additional information from placental and fetal histopathologic assessments and postnatal clinical evaluations”*	Laboratory evidence: ZIKV IgM or IgG in serum samples, if positive ZIKV RNA (RT-PCR) in serum and amniotic fluid offered	Satisfactory
Shiu, C et al. (2018) [34]	Cohort	USA	Jan 2016 – Dec 2016	*“to assess clinical outcomes and challenges associated with Zika virus screening and testing”*	Laboratory evidence: ZIKV RNA (rRT-PCR), ZIKV IgM in serum, placenta or other tissue samples	Satisfactory
Vargas, A et al. (2016) [35]	Case series	Brazil	Aug 2015 – Oct 2015.	*“to describe the first cases of microcephaly possibly related to Zika virus in live born babies reported in the Metropolitan Region of Recife, Pernambuco State, Brazil”*	Presumed infection	Satisfactory
França, G V A et al. (2016) [36]	Case series	Brazil	Nov 2015 – Feb 2016**	*“to describe these newborn babies in terms of clinical findings, anthropometry, and survival”*	Laboratory evidence: ZIKV RNA (RT-PCR) or ZIKV IgM or IgG in serum samples.Presumed infection also acceptable *	Low
Ventura, L O et al. (2017) [37]	Cross-sectional	Brazil	May 2015 – Dec 2015	*“to describe the visual impairment associated with ocular and neurological abnormalities in a cohort of children with congenital Zika syndrome (CZS)”*	Laboratory evidence: ZIKV IgM in cerebral spinal fluid samples	Good

*Presumed infection: when clinical-epidemiological diagnosis were used to determine a ZIKV infection. It can be supported by image data or by discarding other diseases.

** All notified cases in different studies and areas from Brazil during this period are included in this study, i.e., data from Aragão et al., 2017, from December 2015 to February 2016; Krow-Lucal et al., 2018, from November 2015 to February 2016; Brazil et al., 2016, from November 2015 to February 2016; Ventura et al., 2017, November and December 2015.

### Information sources and search strategies

The search strategy aimed to find pertinent data in theses and dissertations in addition to published studies. The following databases were searched by a university librarian on January 8, 2019: MEDLINE via OvidSP (1946 onward), Embase via OvidSP (1947 onward), Cochrane Central Register of Controlled Trials or “Cochrane CENTRAL” via OvidSP (1991 onward), the Cumulative Index of Nursing and Allied Health Literature or “CINAHL” via EBSCOhost (1981 onward), Web of Science Core Collection (1900 onward), ProQuest Dissertations and Theses Global (1861 onward), and Latin American & Caribbean Health Sciences Literature or “LILACS” (1982 onward). The full electronic search strategies for all databases can be accessed via QSpace, Queen’s University’s research repository service [http://hdl.handle.net/1974/24246]. No language or date restrictions were applied. The reference list of systematic reviews and reports were searched for additional studies.

We searched ProQuest Dissertations and Theses and thesis databases from Brazil, Colombia, Canada, USA, and Europe on January 8, 2019, using the terms “zika” or “zikv” or “zyca” or “zyka”. The authors of editorials, correspondence, and conference abstracts that met the inclusion criteria were searched online for original papers.

Additionally, we identified studies that we believed concern PZIK, the prognostic factors of interest, and microcephaly data, but had not published results (i.e. conference abstracts). The first and last authors’ curricula were screened online, and the first authors of each of these studies were contacted. In total, we contacted 17 study investigators at least twice. Responses to seven of the requests were received, all of which informed us of an inability to provide full results.

### Eligibility

We included published data from original research that studied microcephaly as a congenital effect of ZIKV in humans. We included randomized controlled trials, prospective, retrospective or descriptive cohorts, case series (only the case series whose primary data allowed the formation of groups that were prospectively compared regarding outcome), and cross-sectional and case-control studies that could answer the review question.

Studies were excluded if they: (1) were not in humans; (2) did not report our primary objectives; (3) were in vitro/cell studies; (4) were not original research, such as literature reviews, guidelines and manuals, protocol summaries, editorials, opinion pieces, or book chapters; (5) duplicated publications from the same sources (data from the same sources, but published in different papers); or (6) were case reports, epidemiological analyses/bulletins, or case series that included outcomes for only one group.

To avoid double-counting in cases where the same individuals or data were reported in more than one publication, we evaluated publications from the same author or study place (hospital-based, city-based, or state-based population). If there was possible duplication, we used the publication with the most complete information available to extract the data.

### Study selection, data extraction, and assessment of studies

Initial triage of articles was based on the screening of titles and abstracts by two independent evaluators to assess the relevance to the objective of the review. Then, a full-text read of the selected studies was conducted to determine inclusion according to eligibility criteria and the study question. Data extraction and quality assessment were conducted by two independent authors using a standardized instrument. Disagreements at any stage were resolved by consensus or discussion with a third author.

Data extracted included the name of first author, year of publication, period of study, country, language, study design, size, population, and any potential patient-related prognostic factors. These included demography (maternal age, ethnicity, deprivation, education level, marital status, social support); lifestyle factors (smoking habits, drug use); patient history (including comorbidity, family history); symptom type; health care usage (including screening); presenting behaviour; symptom knowledge; and characterization of outcomes.

The methodological quality and risk of biases in the studies were assessed by two independent reviewers using the Newcastle-Ottawa Quality Assessment Scale (NOS) [38]. The studies that received 7 stars or more were considered to be of high quality, those that received 6–5 stars were considered to be of satisfactory quality, and those with 4 or less stars were considered to be of low quality [39].

### Synthesis of results and summary measures

To ensure comparable and accurate results to perform the meta-analysis, we contacted the corresponding authors of all the included studies in the systematic review up to three times to ask whether they would be willing to provide results. A standardized table (Additional Table 2) was sent to all the authors to complete using their primary data, considering as a population of interest only cases of PZIK, and the outcome of interest being the presence or absence of microcephaly.

**Table 2:** Population characteristics of the studies included in the meta-analysis

Study	# enrolled ZIKV+ pregnant women	# enrolled ZIKV+ newborns/fetuses	# ZIKV+ newborns/fetuses with microcephaly	Sex (male/total)		Maternal ethnicity (%)		Maternal age – mean (SD)
				Microcephaly +	Microcephaly -	Microcephaly +	Microcephaly -	
Aragão, MFVV et al. (2017) [26]	U*	19	16	–	–	–	–	
Schaub, B et al. (2017) [27]	14	14	9	5/9	4/3	9/9	–	26.78 (6.33)
Krow-Lucal, ER et al. (2018) [28]	U*	115	43	–	–	–	–	
Honein, M A et al. (2017) [29]	442	55	18	–	–	–	–	
Kumar, M et al. (2016) [30]	4	4	3	1/3	0/1	3/3	1/1	27 (5.57)
Brasil, P et al. (2016) [31]	134	134	4	–	–	–	–	
Pomar, L et al. (2017) [32]	301	278	28	15/28	126/250	27/28	244/250	28.08 (7.75)
Sanz Cortes, M et al. (2018) [33]	12	9	7	–	–	–	–	
Shiu, C et al. (2018) [34]	8	87	5	–	–	–	–	
Vargas, A et al. (2016) [35]	U*	40	40	20/43	5/14	12/43	2/14	23.5 (8)
França, G V A et al. **(2016) [36]	1501	602	330	244/567^!^	221/691^!^	495/567^!^	591/691^!^	24.79 (6.668)
Ventura, L O et al. (2017) [37]	U*	32	29	54/148	9/148	–	–	27.36 (7.28)

* Unknown (not reported in the paper)

** All notified cases in different studies and areas of Brazil during this period are included in this study, i.e., data from Aragão et al., 2017 from December 2015 to February 2016; Krow-Lucal et al., 2018, from November 2015 to February 2016; Brazil et al., 2016, from November 2015 to February 2016; Ventura et al., 2017, November and December 2015

We asked about maternal and infant data, considering the following variables: population size (number of newborns/fetuses); maternal age; maternal ethnicity; maternal schooling; maternal symptoms compatible with ZIKV infection during gestation; maternal smoking habits and/or use of alcohol and/or other drugs; presence of comorbidities in the mother; trimester of pregnancy when infection occurred; prior yellow fever vaccine exposure; prior exposure to other vaccines; fetus sex; and newborn gestational age at birth. The results provided by the authors were also used to complement the description of the studies in the qualitative synthesis.

Since the characterization of microcephaly can vary among countries, for the meta-analysis we used the definition recommended by WHO: the measure of the head circumference (HC) of less than two Standard Deviations (SD) below the average for sex and gestational age [40]. Also, as a reliable laboratory diagnostic test of ZIKV was not available during the first part of the outbreak, we used the ZIKV case definition as reported by the study. All the authors were advised to follow these criteria when providing the data.

Meta-analyses were performed if sufficient results were available. Summary measures [Relative Risk (RR) and Odds Ratio (OR)] were calculated for each different variable included. The random effects model was used when the I^2^ test demonstrated a large degree of heterogeneity between studies (I^2^ higher than 0.5). When the heterogeneity between studies was equal or lower than 0.5, fixed-effect model was used for the analysis. We used the RevMan software to execute the calculation of summary measures and heterogeneity of the studies, and to generate the figures of the meta-analysis (Forest plots).

## Results

### Characteristics of included studies

**Fig. 1:**
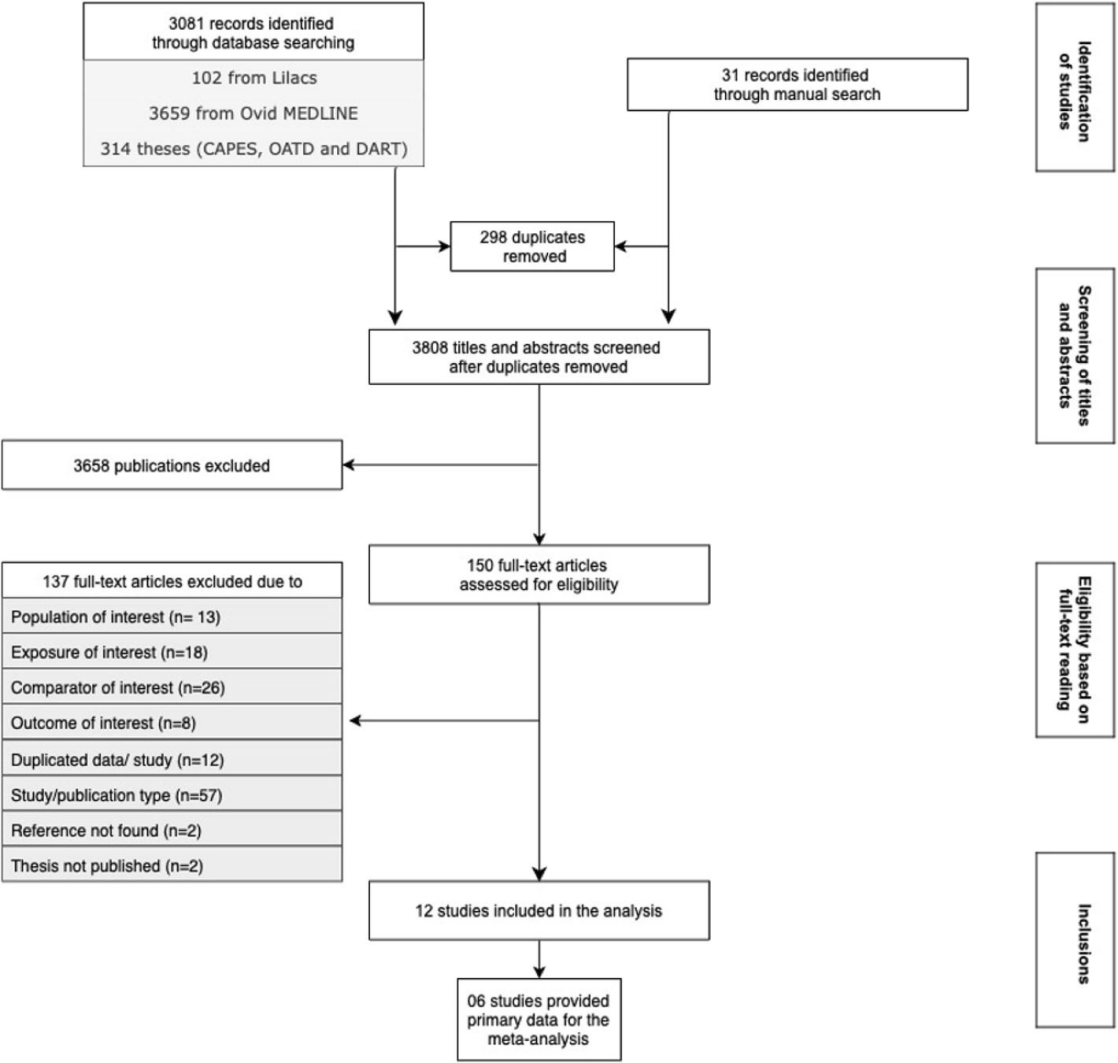
Flow diagram of Selection of studies for the systematic review and meta-analysis

Our systematic review identified 4106 records, from which 298 duplicates were removed and 3808 titles and abstracts were screened. After this process, considering the eligibility criteria, the reviewers identified 150 unique studies that seemed to be relevant to our question, and those articles were assessed in full-text for eligibility. From those, 138 studies were ruled out based on the exclusion criteria. After the full-text review, a total of 12 observational studies were included in the synthesis of the literature [26–37] (Fig. 1). Of the 12 eligible studies, six authors provided the primary data [27, 30, 32, 35–37], and therefore were included in the meta-analysis. This process is summarized in Fig. 1.

The twelve included studies were published between 2015 and 2018; ten of them had Research Ethics Board approval and two declared themselves exempt from this approval [29, 35]. Five were cohort studies [29, 31–34], three were case-controls studies [26, 28, 30], three were case-series [27, 35, 36], and one was a cross-sectional study [37]. The studies defined as case-series [27, 35, 36] were conducted based on surveillance data – local surveillance system and hospital-based surveillance – and reported only ZIKV infected cases with varied outcomes. For that reason, as it was possible to compare the microcephaly group and the non-microcephaly group, for the meta-analysis, we considered them as “prospective observational studies” in the same group as the cohort studies of PZIK.

All 12 studies were conducted in the Americas: three of them in the United States [29, 30, 34], one in the Caribbean [27], and eight in South America, i.e., Brazil [26, 28, 31, 35–37], French Guiana [32], and Colombia [33]. The total population enrolled in the included studies is 6154 newborns/fetuses. From those, 1120 (18.20%) had a diagnosis of ZIKV infection, of whom 509 (45.45%) were diagnosed with microcephaly. Most of the studies [26, 28–32, 34, 35, 37] addressed the link between PZIK and neurological findings or other outcomes in newborns/fetuses; however laboratory-confirmed ZIKV infection was not consistent across all the studies.

The assessment of PZIK varied among studies. One of the included studies [35] also included women who did not undergo a laboratory test to determine ZIKV infection. In this study, ZIKV infection was defined based on epidemiological link and clinical characteristics, as accepted by the Ministry of Health. Regarding laboratory evidence, six tested the mothers/pregnant women [30, 31, 33–36], and six studies tested both mothers/pregnant women and newborns/fetuses [26–29, 32, 37] (Table 1). RT-PCR was used in seven studies [27, 29, 31–34, 36] in at least one phase of the diagnosis, but only two of them used this test as the confirmation tool for all the cases [27, 31]. The plaque reduction neutralization test (PRNT) was used in five studies [26, 28, 29, 32, 34] and 10 used serological testing to detect either IgG or IgM antibodies [26–30, 32–34, 36, 37].

Microcephaly definitions varied across studies, changing over time. Regarding the moment of detection, microcephaly was diagnosed after delivery in all the 12 studies, but three of them [27, 31, 33] also performed fetal ultrasounds to detect microcephaly.

Based on the NOS, four studies [29–31, 37] were deemed to be of good quality and seven [26–28, 32–35] were of satisfactory quality (Additional Table 3, 4, 5 and 6). We summarize the characteristics of all the 12 selected studies in Table 1 and the characteristics of the population enrolled in Table 2.

Regarding the quantitative synthesis, six authors provided the primary data that was used in the meta-analysis. Of these, three [32, 35, 36] were cohort studies (total *N* = 1593), two [27, 30] were case-controls (total *N* = 18), and one study with 32 cases was cross-sectional [37]. The total number of newborns/fetuses that had microcephaly were 638 (40.05%) in the prospective studies and 12 (85.71%) in the retrospective studies. Ventura et al. [37] also provided the primary data, but since it is the only cross-sectional study, we did not include these results in the meta-analysis. It was not possible to explore publication bias, as less than four included studies used the same methodology, most of them with small sample sizes.

### Microcephaly

The 12 selected studies reported a higher risk of microcephaly with the presence of ZIKV infection during gestation, with an Odds Ratio as high as 21.9 (95% Confidence Interval – CI of 7. 0, 109.3) [28] and a Relative Risk of 6.63 (95% CI, 0.78, 57.83) [32] when compared to no ZIKV infection during gestation. When analysing only cases with ZIKV infection during gestation, microcephaly was prevalent in up to 54.82% of the infants enrolled in one study [36]. Considering the 705 newborns/fetuses diagnosed as ZIKV positive and whose mothers had symptoms of ZIKV infection during pregnancy described in the published papers [24–29, 32, 33, 38–40], we found a prevalence of microcephaly of 52.63% (CI95% = 48.3, 56.95) in the symptomatic group versus a prevalence of microcephaly of 45.64% (CI95% = 41.02, 50.26) in the asymptomatic group.

Schaub et al. [27] reported 14 cases of ZIKV infection during gestation. They found microcephaly in nine (64.28%) of them. But only one of the pregnancies resulted in a live birth (born at 40 weeks with microcephaly), with one case of intra-uterine death at 25 weeks. The 12 other cases had a termination of pregnancy varying from 18 weeks and 3 days to 34 weeks of gestation. For that reason, the data on “gestational age at birth” of this study was not included in the analysis.

### Assessed prognostic factors

The symptoms of ZIKV infection during pregnancy were assessed in all studies except in Kumar et al. [31], which performed laboratory analyses of stored plasma samples from mothers who gave birth to microcephalic and healthy babies, collected before ZIKV was linked with microcephaly. Overall, symptoms of ZIKV infection during gestation were present in 705 of the 1116 pregnant women infected (63.17%). From the studies with this available information [26, 28, 31–33, 35–37], 270 of the 513 women who reported symptoms during pregnancy (52.63%), delivered an infant with microcephaly.

The trimester of pregnancy when the infection occurred was assessed in eight studies [26, 29, 31–33, 35–37]. From the cases of ZIKV infection during the first trimester (*n* = 324), 42.59% exhibited microcephaly. Among those with ZIKV infection during other stages of pregnancy [second trimester (*n* = 332) and third trimester (*N* = 141)], 21.99% exhibited microcephaly.

Sanz Cortes et al. [33] and Schaub et al. [27] reported maternal nutritional status [mean maternal Body Mass Index (BMI) of 24.38 kg/m2 (SD 5.56) and 26.54 kg/m2 (SD 5.76), respectively]. Sanz Cortes et al. reported the mean maternal BMI in the microcephaly group and non-microcephaly group as 25.88 kg/m2 (SD 3.83) and 19.89 kg/m2 (SD 8.43), respectively [33]. Schaub et al. reported mean maternal BMI as 27.84 kg/m2 (SD 6.77) in the microcephaly group and 24.20 kg/m2 (SD 2.39) in the non-microcephaly group [27].

Although Vargas et al. [35] measured all the variables of interest, they mentioned that five (8.3% of the total population) cases of microcephaly were due to other congenital infections and did not explore the data separately. For that reason, it was not possible to use their data for analysis of PZIK.

Concerning comorbidities, other infections were excluded in most of the studies (7/12). Infections known to have teratogenic effects, such as syphilis, toxoplasmosis, rubella, cytomegalovirus, and herpes simplex (STORCH). were excluded in six studies [27, 31–33, 36, 37]; dengue virus infection in four [27–29, 31]; HIV in four [27, 31, 33, 37]; chikungunya in two [24, 40]; parvovirus in one [31]; and other sexually transmitted infections in one [33].

Three studies [31, 33, 37] provided information regarding the consumption of licit or illicit substances during pregnancy. Sanz Cortes et al. [33] used these exposures as exclusion criteria, Brasil et al. [31] informed that all included women reported no medication use, and Ventura et al. [37] reported four cases (12.5%), all of them in the microcephaly group (13.79% of the microcephaly outcomes), but did not mention which particular substance was assessed.

Three studies reported the presence of singleton versus multiple gestation [29, 31, 32] and the delivery method among the PZIK cases was reported in two studies. Brasil et al. (2017), reported a C-section rate of 82.4% (*N* = 89/108) and Sanz Cortes et al. (2018) of 66.67% (*N* = 6/9).

Gestational age at birth in newborns with microcephaly due to PZIK was not provided in four studies [28, 29, 35, 36]. One study [27] had only one newborn (7%), 40 weeks at birth, while all the other analysed cases (13 cases, 93%) had a termination of pregnancy at different times of the gestational outcome. Aragão et al. [26] and Sanz Cortes et al. [33] presented the mean and SD of all the population enrolled, finding 36.29 (SD 8.71) weeks of gestation and 37.8 (SD 1.15) weeks of gestation, respectively. Brasil et al. [31] provided the gestational age at birth of the 58 (43.3%) participants who had any abnormal finding at birth. Of these, four (6.9%) were in the microcephaly group, two of which were born preterm. From the non-microcephaly group, five (11.63%) newborns were born preterm. Shiu et al. [34] reported data of 86 women with laboratory evidence of PZIK. They did not provide the data regarding the presence or absence of microcephaly, but 34 (39.5%) of the mothers were still pregnant by the time of the report, eight (9.3%) had preterm delivery, and 44 (51.1%) had term delivery. From the remaining population (*n* = 314) [30, 32, 37], the studies provided mean and SD. The weighted mean and SD in the microcephaly group (*n* = 60) was 37.91 (SD 2.72) gestational weeks at birth and that of the infants in the non-microcephaly group was 38.06 (SD 2.42).

### Studies that provided data to conduct the meta-analysis

Six studies provided primary data to perform the meta-analysis [27, 30, 32, 35–37]. Three of them [32, 35, 36] had prospective designs, two [27, 30] had retrospective designs, and one [37] was a cross-sectional study. The study by Ventura et al. [37] was the only cross-sectional one, and therefore, we were not able to incorporate the data into the meta-analysis. For this reason, we describe it briefly, below.

Ventura et al. [37] provided data on 148 cases of PZIK. Microcephaly status (presence or absence) was reported for 140 (94.6%) of these infants. Of these, 124 (88.6%) presented microcephaly. In the microcephaly group, 56.45% (*n* = 70) were female, while in the non-microcephaly group, the majority were male (*n* = 9/16, 56,25%). The information on maternal symptomatology of PZIK was available for 132 infants (116 with microcephaly). The mothers of 108 of the infants reported symptoms (such as rash, pruritus, and conjunctivitis), 97 (83.6%) of whom belonged to the microcephaly group. Data on the use of licit or illicit substances was available for 132 mothers (118 in the microcephaly group) and 13 of them reported the use of these substances. In the microcephaly group, 12 (10.2%) mothers reported this behaviour, and in the non-microcephaly group, one mother (7.1%) reported it. As for the gestational trimester of infection, most of the mothers in the microcephaly group were infected in the first trimester (*n* = 48, out of 100 with this information available), and the majority (*n* = 7, out of 13 with this information available) in the non-microcephaly group had ZIKV infection in the second trimester. There was not enough data on maternal schooling and previous vaccines (yellow fever or other) to conduct an analysis. Regarding the methodological quality, this study was assessed as good quality.

### Meta-analysis

We conducted meta-analysis to assess the rate of microcephaly detection according to seven identified characteristics: (i) sex (proportion of males); (ii) maternal age; (iii) maternal ethnicity (proportion of non-white); (iv) gestational age at the birth; (v) presence of symptoms during gestation; (vi) presence of comorbidities; (vii) gestational trimester of infection; and (viii) smoking habits and/or alcohol or other drug consumption.

**Fig. 2:**
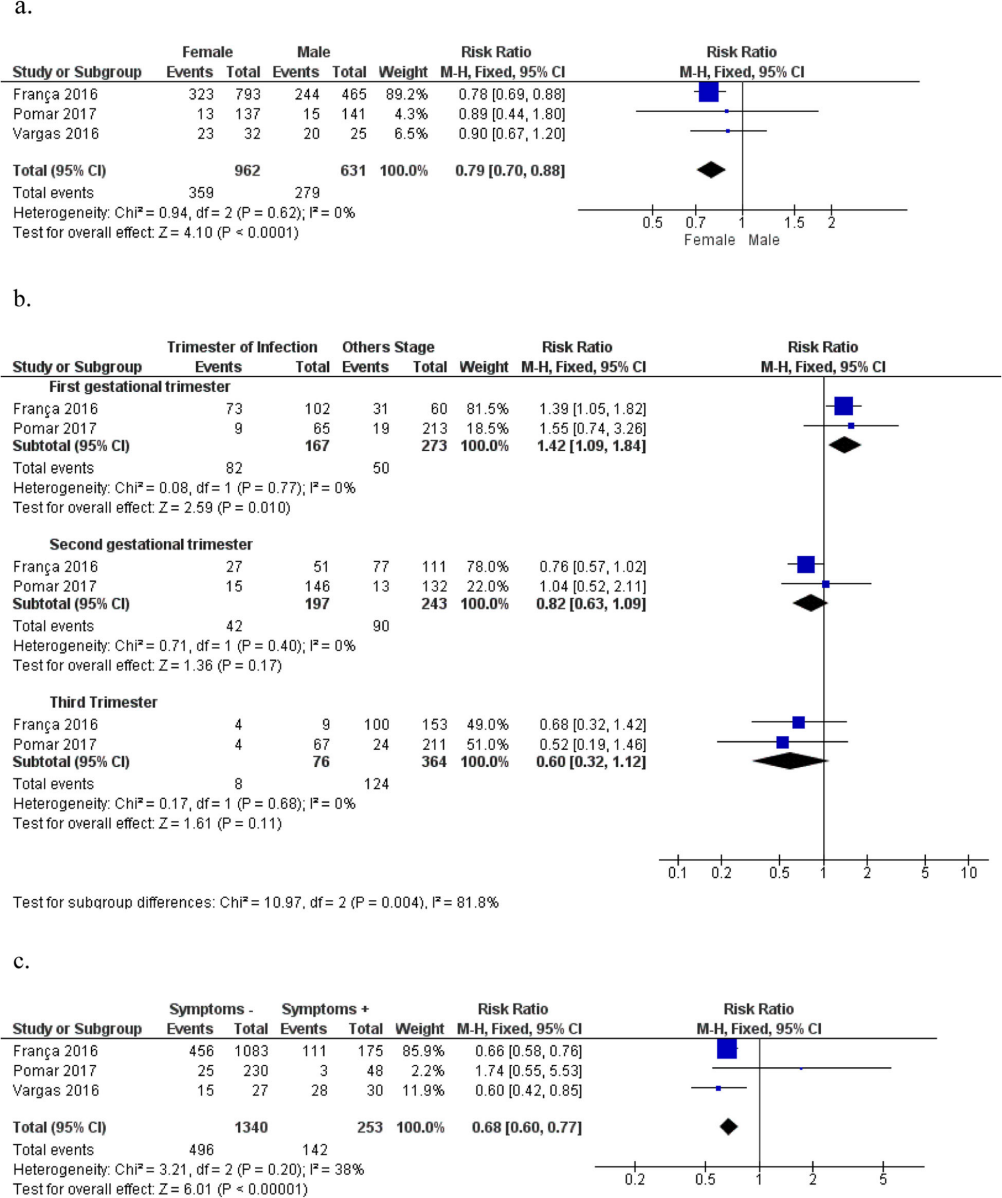
Meta-analysis forest plot for prospective studies. 2a. Sex of the newborns/fetuses. 2b. Trimester of pregnancy when ZIKV infection occurred. 2c. Symptoms of ZIKV infection during pregnancy

Regarding the meta-analysis of prospective studies, only three variables showed to be significant (presented in Fig. 2). In relation to the sex of newborns/fetuses, females presented a lower risk of microcephaly compared to males (RR 0.79; 95% CI 0.70, 0.88; I^2^ = 0%) (Fig. 2a). Infection in the first trimester of pregnancy (Fig. 2b) was a risk factor (RR 1.42; 95% CI 1.09, 1.84, I^2^ = 0%) for microcephaly, compared to infection in the second and third trimesters of pregnancy. A decrease in the microcephaly detection risk rate was observed in women who did not presented symptoms of PZIK (RR 0.68; 95% CI 0.60, 0.77; I^2^ = 38%), such as conjunctivitis, pruritus, and rash (Fig. 2c).

There was no statistically significant difference between groups regarding maternal ethnicity – white (RR 0.91; 95% CI 0.77, 1.08; I^2^ = 0%) – or the absence of tobacco, alcohol, and/or other substance consumption (RR 0.84, 95% CI 0.55, 1.29, I^2^ = 0%), although the point estimates indicated these characteristics as probable protective factors. Maternal age and gestational age at birth – analysed using mean and SD – were also similar between groups. The meta-analysis data of the factors that did not significantly increase the risk are illustrated in the Additional Fig. 1.

As to the methodological quality of the prospective studies included in the meta-analysis, França et al. [36] was the only included study assessed as low quality. Pomar et al. [32] and Vargas et al. [35] were considered as satisfactory quality.

In relation to the retrospective studies [27, 30], Kumar et al. [30] tested archived blood samples collected at delivery at the Kapiolani Medical Center for Women and Children in Hawaii; and Schaub et al. [27] investigated 12 cases diagnosed during pregnancy, with only one live birth and 11 cases terminated in pregnancy. Only the infants’ sex could be tested as an exposure factor in the retrospective study design. There was a decrease of the Odds Ratio (OR) of microcephaly in females, although it was not significant (OR 0.54; 95% CI 0.08, 3.66, I^2^ 0%) (Additional Fig. 2a). It was not possible to analyse the data on trimester of infection, presence of symptoms, substance consumption, and vaccine exposure, as only one study had this data available. Maternal age, maternal ethnicity, and presence of comorbidities were not estimated, as the study by Kumar et al. [30] had only one case without microcephaly and Schaub et al. [27] included only non-white individuals without comorbidities (Additional Fig. 2b). Regarding quality assessment, Kumar et al. [30] was assessed as good methodological quality and Schaub et al. [27] as satisfactory methodological quality.

## Discussion

To our knowledge, this is the first systematic review to evaluate maternal and fetal prognostic factors that may contribute to the presence of microcephaly in newborns and fetuses when the mother was infected with ZIKV during pregnancy. Our meta-analysis showed that infection in the first trimester of pregnancy may increase the risk of microcephaly by 41% when compared to other trimesters, and female fetuses have a lower risk of developing microcephaly. Our study did not show differences between groups regarding maternal age, ethnicity, presence of comorbidities, and consumption of alcohol or other substances during gestation.

In relation to trimester of infection, other STORCH infections also confer differential risk of congenital defects according to the stage of pregnancy in which infection occurs [41–44]. These events are related to both the development of the central nervous system (CNS) and the fetus immune response [45]. Even though ZIKV infection in the second and third trimester of pregnancy seemed to be a lower risk compared to ZIKV infection in the first trimester, it is important to highlight that this infection carries a risk for the development of microcephaly and other adverse pregnancy outcomes throughout the full duration of pregnancy [4, 46, 47]. There is still a lack of knowledge on the magnitude of the risk of newborns infected by ZIKV developing microcephaly later in childhood [48, 49].

The relationship between fetal gender and adverse pregnancy outcome is controversial [50, 51]. The male sex, especially in low-risk pregnancies, seems to have an effect on adverse pregnancy outcomes [52] such as preterm births [53, 54] and stillbirths [55]. During the ZIKV outbreak on Yap Island, a higher prevalence of IgM antibody against ZIKV was found in men as compared to women [15]. It should be pointed out that our meta-analysis (point estimates) of both prospective and retrospective studies showed that females had a lower risk of developing microcephaly than males. However, the OR was not significant (0.54, IC95% 0.08, 3.66), probably due to the small number of individuals included in the retrospective studies in the meta-analysis. Additionally, the studies of Pomar et al. [32] and Vargas et al. [35], both of satisfactory quality, did not show a statistically significant relation. Nonetheless, our findings reinforce previous studies that support a male-biased incidence in infectious diseases, [51, 56] pointing out a probable relationship between microcephaly and fetal sex, with males being at a higher risk than females.

It is important to stress that the symptoms of ZIKV infection, both in men and women, are often mild and infrequent [4, 15, 57, 58]. There is still uncertainty about whether symptoms can be addressed as reliable indicators of vertical transmission or disease severity [59–61]. Other infections that lead to congenital malformation, such as cytomegalovirus, also have a high number of asymptomatic cases, but, when present, symptoms might indicate an adverse outcome [62].

Our meta-analysis results suggest an association of microcephaly with symptoms, probably restricted by the heterogeneity of the studies. Even so, this association may be influenced by recruitment and selection, given that tree studies [27, 36, 37] performed the recruitment based only on the infants, thus increasing the microcephaly rate compared to the microcephaly rate observed in studies that included all pregnant women infected with ZIKV. Also, asymptomatic ZIKV infection in pregnant women could decrease the sensibility of the microcephaly detection, specifically in areas where ZIKV surveillance was inadequate. Furthermore, two studies [35, 36] used cases of presumed ZIKV infection that were diagnosed based on clinical-epidemiological evidence and not laboratory tests. In this sense, although the low viremia induced by ZIKV infection increases false negative results [63], the most reliable diagnostic test is RT-PCR in the newborn sample.

Regarding socioeconomic, demographic, and environmental factors linked to adverse pregnancy outcomes such as maternal ethnicity, low family income, maternal schooling, and maternal age, this review was unable to determine if these can act as additional prognostic factors in microcephaly development, as only a few of the included studies assessed them. The risk ratio – point estimates of maternal ethnicity (non-white, RR 0.91, CI 95% 0.77, 1.08) and the absence of alcohol, tobacco, and/or other substance consumption during pregnancy (RR 0.84, CI 95% 0.55, 1.29) suggest that these are protective factors for microcephaly, yet differences were not statistically significant.

Our results suggest that maternal age and ethnicity are not prognostic factors for microcephaly. On the other hand, the presence of comorbidities and substance consumption during pregnancy may have been influenced by the small sample size of the studies included in the meta-analysis, restricting our results, since those factors have been reported in the literature as being capable of interaction with other prognostic factors increasing the risk of adverse outcomes [64–67].

The inconsistency between the studies also influenced the analysis concerning health factors such as microcephaly history in the family, maternal comorbidities, and nutritional status. Nevertheless, our review was able to indicate that the presence of comorbidities might raise the risk of microcephaly, although we were not able to find statistically significant differences. The presence of antibodies for other flaviviruses such as dengue and yellow fever, which may act as modulators of adverse outcomes, as well as prior exposure to the yellow fever vaccine, were not explored in most of the studies, thus hampering the analysis of possible effect modifiers. This is likely related to the urgency of assessing the relationship between PZIK and intergenerational effects [68–70] at the time when most of the studies were conducted.

Regarding the methodological quality of the studies included in the meta-analysis, França et al. [36] was the only low-quality study, but because of the size of its population, this study received the highest weight among the prospective studies included. This study used secondary data and was able to collect information on 1258 ZIKV-infected pregnant women – the highest number of individuals in the included studies. In the same perspective, Pomar et al. [32] was deemed to be as of satisfactory quality and included 278 ZIKV-infected participants. Their confidence interval was often large, as it included the estimates found in the other studies, reducing the I^2^ of the meta-analysis of prospective studies. On the other hand, Kumar et al. [30], a retrospective study, included four cases and was assessed as good quality. This study also had a large confidence interval, incorporating the estimates of Schaub et al. [27], therefore reducing the I^2^.

The complexity of the prognostic factors associated with microcephaly due to ZIKV infection during pregnancy and the broader socioeconomic context in which it occurs, including an increased social and economic impact caused by the Congenital Zika Syndrome, must be considered when designing preventive programs or providing health care. Although this review and meta-analysis only approached individual-level factors, the most appropriate interventions might be on the ecological level, especially in low-income countries addressing pathways of infection by mosquito control and protective measures against sexual transmission.

### Limitations of this systematic review

Common sources of bias in any meta-analysis are publication bias and heterogeneity between studies. We assessed the publication bias by reviewing the grey literature, looking for recent published manuscripts by authors who published theses and presented conference abstracts, and contacting them. Despite this effort, no additional data were found. Thus, the meta-analysis only included published studies. Because of the small number of included studies, we were not able to perform tests to detect publication bias. Since nonsignificant results have a decreased likelihood of publication, we believe that the included studies might be reporting a higher association between PZIK and birth defects than may generally be the case. However, in terms of associated prognostic factors for the development of microcephaly – our exposures of interest –, it is unlikely that publication bias would affect our results. Regarding summary measures, we understand that our data also reflect the number of participants in each study (and not the methodological quality of them), as is observed in all unweighted summary measures.

Concerning the design of the studies, the case-series studies did actually include the entire available populations, using surveillance strategies, tending to have the characteristics of a descriptive cohort study. However, due to the small number of participants, they were designed and assessed as case-series. Moreover, the small number of individuals included in the retrospective studies restricted the power of the meta-analysis.

Specific limitations of our review were the non-inclusion of in vitro studies in the eligibility criteria – some authors have reported that the ZIKV strain can be related to varied outcomes [71, 72] –, and the inherent differences between studies, especially in regard to the ZIKV infection definition and data collection. There is still a lack of consensus on diagnostic strategies for ZIKV [63], and the studies identified in this review used different tools for this purpose. Also, as cited, most of the studies did not assess the possible effect modifiers that this review was seeking to analyse.

It is necessary to point out that the systematic review might have some duplication of cases, as the study of França et al. [36] included all the notified cases in Brazil from October 2015 to February 27th, 2016. Other studies using this time frame may have used individuals as they were notified in the national system. However, the data used in the meta-analysis does not overlap in time or place, so this was not considered a hurdle for our results.

Our study design was formulated in such a way that we would not exclude any study after its quality assessment. The methodological quality of included studies was considered predominantly satisfactory and four studies were assessed as good quality [28–30, 37]. The only study [36] that scored as low quality had the largest population and, therefore, the smallest confidence intervals. Although the weight of the study may have influenced our summary measures, the estimate of the other prospective studies [32, 35] went in the same direction, reinforcing our conclusions.

Finally, our findings are supported by observational studies only. The small number of included studies reflected a lack of adequate studies in the literature for the investigation and understanding of the prognostic factors related to the association of microcephaly and congenital zika infection. Additionally, since there were different case definitions for ZIKV infection across the studies and laboratory tests are still not fully reliable, the studies may have included non-ZIKV infections in the analysed groups, therefore introducing a possible measurement bias which could sway our results to either side, but most probably towards a null hypothesis. For these reasons, our results should be interpreted cautiously so as not to influence prenatal care or health surveillance strategies used to detect and prevent new cases.

## Conclusions

This systematic review and meta-analysis found that the male sex, the occurrence of ZIKV infection in first trimester, and symptomatic infection increase the risk of microcephaly. The available evidence does not establish maternal age and ethnicity as prognostic factors. The prognostic effect of previous antibodies for other flaviviruses, family history of microcephaly or other congenital abnormality, family income, schooling level, and civil status remain unclear. These findings should be interpreted cautiously because ZIKV is an emergent disease and its effects are still under study. Still, they can be used to reduce false alarms regarding maternal age and ethnicity as prognostic factors; to increase preventive strategies to ZIKV infection, especially in the first trimester; and to understand that, due to the lack of reliable diagnostic tests, specifically after the viremic period, the presence of symptoms may be a good indicator of ZIKV infection, and that pregnant women reporting them should receive more attentive antenatal care.

This study only reviewed prognostic factors for microcephaly related to ZIKV, but the effects of Congenital Zika Syndrome is inconstant and other factors may be associated with the different outcomes of ZIKV infection during pregnancy. Although there has been a high demand for and a high production of studies to understand the pathogenicity of ZIKV in the last three years, the studies conducted show a high heterogeneity in both methods and data collection. This highlights the need for dialogue between researchers seeking to investigate an emergent problem in public health. Future research needs to homogenize definitions of relevant outcomes, test hypotheses of potential disease modulators, include other aspects of the Congenital Zika Syndrome other than microcephaly, and include other variables related to birth defects. 

## Supplementary information

**Additional file 1 Additional** Table 1**.** PRISMA Checklist.

**Additional file 2 Additional** Table 2**.** Standardized table sent to all the corresponding authors.

**Additional file 3 Additional Table 3.** Newcastle-Ottawa Quality Assessment Scale - retrospective studies.

**Additional file 4 Additional Table 4.** Newcastle-Ottawa Quality Assessment Scale - cohort studies.

**Additional file 5 Additional Table 5.** Newcastle-Ottawa Assessment Scale adapted for cross-sectional studies.

**Additional file 6 Additional Table 6.** Newcastle-Ottawa Assessment Scale adapted for case reports and case series .

**Additional file 7 Additional Fig. 01.** Meta-analysis forest plot of prospective studies. **Additional Fig. 01a.** Maternal ethnicity. **Additional Fig. 01b.** Smoking habits and/or consumption of alcohol and/or other substances during pregnancy. **Additional Fig. 01c.** Maternal comorbidities during pregnancy. **Additional Fig. 01d.** Mean maternal age. **Additional Fig. 01e.** Mean gestational age at the birth.

**Additional file 8 Additional Fig. 02.** Meta-analysis forest plot of retrospective studies. **Additional** Fig. 02a**.** Sex of newborns/fetuses in case-control studies. **Additional Fig. 02b.** Maternal comorbidities during pregnancy.

## Data Availability

The data supporting the findings of this analysis, as well as data not included in it, are available from the corresponding author. However, restrictions apply to the availability of these data, which were used under licence for the current study, and therefore, are not publicly available. Data are available from the corresponding author upon reasonable request.

## Abbreviations

CI 95%: Confidence Intervals of 95%
CNS: Central nervous system
CZS: Congenital Zika Syndrome
NOS: Newcastle-Ottawa Quality Assessment Scale
OR: Odds ratio
PZIK: Zika virus infection during pregnancy
RR: Relative risk
SD: Standard deviation
STORCH: Syphilis, toxoplasmosis, rubella, cytomegalovirus, and herpes simplex
WHO: World Health Organization
ZIKV: Zika virus

## References

[1] WHO. WHO statement on the first meeting of the International Health Regulations (2005) (IHR 2005) Emergency Committee on Zika virus and observed increase in neurological disorders and neonatal malformations [Internet]. Statement. 2016 [cited 2016 May 20]. Available at: http://www.who.int/en/news-room/detail/01-02-2016-who-statement-on-the-first-meeting-of-the-international-health-regulations-(2005)-(ihr-2005)-emergency-committee-on-zika-virus-and-observed-increase-in-neurological-disorders-and-neonatal-malformations.

[2] Krauer F, Riesen M, Reveiz L, Oladapo OT, Martinez-Vega R, Porgo T V, et al. Zika Virus Infection as a Cause of Congenital Brain Abnormalities and Guillain-Barré Syndrome: Systematic Review. Plos Med [Internet]. 2017 Jan;14(1):e1002203. Available at: http://ovidsp.ovid.com/ovidweb.cgi?T=JS&CSC=Y&NEWS=N&PAGE=fulltext&D=emexa&AN=614265070. 10.1371/journal.pmed.1002203 PMC5207634 28045901

[3] Wang J-N, Ling F. Zika Virus Infection and Microcephaly: Evidence for a Causal Link. Int J Environ Res Public Health [Internet]. 2016 Oct;13(10):1031. Available at: http://ovidsp.ovid.com/ovidweb.cgi?T=JS&CSC=Y&NEWS=N&PAGE=fulltext&D=emexa&AN=612835643. 10.3390/ijerph13101031 PMC5086770 27775637

[4] Chibueze EC, Tirado V, Lopes K, Da S, Balogun OO, Takemoto Y, Swa T, et al. Zika virus infection in pregnancy: a systematic review of disease course and complications. Reprod Health [Internet]. 2017 28 [cited 2017 Sep 5];14(1):28. Available at: http://ovidsp.ovid.com/ovidweb.cgi?T=JS&CSC=Y&NEWS=N&PAGE=fulltext&D=emexa&AN=616827641. 10.1186/s12978-017-0285-6 PMC5330035 28241773

[5] de Araujo TVB, Ximenes RAA, Miranda-Filho DB (2018). Association between microcephaly, Zika virus infection, and other risk factors in Brazil: final report of a case-control study (vol 18, 2017). Lancet Infect Dis.

[6] Rasmussen SA, Jamieson DJ, Honein MA, Petersen LR. Zika virus and birth defects - Reviewing the evidence for causality. N Engl J Med [Internet]. 2016 19 [cited 2016 Aug 10];374(20):1981–7. Available at: http://ovidsp.ovid.com/ovidweb.cgi?T=JS&CSC=Y&NEWS=N&PAGE=fulltext&D=emed18&AN=610738819. 10.1056/NEJMsr1604338 27074377

[7] Oliveira Melo AS, Malinger G, Ximenes R, Szejnfeld PO, Alves Sampaio S, Bispo de Filippis AM. Zika virus intrauterine infection causes fetal brain abnormality and microcephaly: tip of the iceberg? Ultrasound Obstet Gynecol [Internet]. 2016 Jan [cited 2016 Aug 27];47(1):6–7. Available at: http://doi.wiley.com/10.1002/uog.15831. 10.1002/uog.15831 26731034

[8] Pomar L, Musso D, Malinger G, Vouga M, Panchaud A, Baud D. Zika virus during pregnancy: From maternal exposure to congenital Zika virus syndrome. Prenat Diagn [Internet]. 2019 [cited 2019 Sep 30];39(6):420–30. Available at: http://www.ncbi.nlm.nih.gov/pubmed/30866073. 10.1002/pd.5446 30866073

[9] Peñas J, Andújar F. Alteraciones del perímetro craneal: microcefalia y macrocefalia. Pediatr Integr [Internet]. 2003 [cited 2016 Aug 23]; Available at: http://acondroplasiauruguay.org/documentos/informacion medica/a/Perimetro craneal macrocefalia.pdf.

[10] Wiwanitkit V (2017). Microcephaly and Zika virus Infection. J Craniofac Surg [Internet].

[11] Brazil. Monitoramento integrado de alterações no crescimento e desenvolvimento relacionadas à infecção pelo vírus Zika e outras etiologias infecciosas, até a Semana Epidemiológica 20 de 2018. Bol Epidemiológico | Secr Vigilância em Saúde | Ministério da Saúde [Internet]. 2018 [cited 2019 Jan 7];49(54):8. Available at: http://portalarquivos2.saude.gov.br/images/pdf/2018/dezembro/14/2018-061.pdf.

[12] Kleber de O, Araújo de F, Carmo EH, Duncan BB de SK, Inês Schmidt M, et al. Infection-related microcephaly after the 2015 and 2016 Zika virus outbreaks in Brazil: a surveillance-based analysis [Internet]. Vol. 390 North, Lancet. Secretariat of Health Surveillance, Brazilian Ministry of Health, Brasilia, Brazil.; Postgraduate Program in Epidemiology, Universidade Federal do Rio Grande do Sul, Porto Alegre, Brazil.; Secretariat of Health Surveillance, Brazilian Ministry of Health, : Lancet; Lancet; 2016. p. 861; 1051–870; 1051. Available at: http://proxy.queensu.ca/login?url=http://search.ebscohost.com/login.aspx?direct=true&db=cin20&AN=124976724&site=ehost-live. 10.1016/S0140-6736(17)31368-5 28647172

[13] Cauchemez S, Besnard M, Bompard P, Dub T, Guillemette-Artur P, Eyrolle-Guignot D (2016). Association between Zika virus and microcephaly in French Polynesia, 2013-15: A retrospective study. Lancet [Internet].

[14] Hawaii. Hawaii Birth Defects Surveillance Report [Internet]. 2011 [cited 2019 Oct 1]. Available at: https://health.hawaii.gov/genetics/files/2013/04/HBD_Surveillance_Report_1986-2005.pdf.

[15] Duffy MR, Chen T-H, Hancock WT, Powers AM, Kool JL, Lanciotti RS, et al. Zika virus outbreak on Yap Island, Federated States of Micronesia. N Engl J Med [Internet]. 2009 11 [cited 2015 Dec 9];360(24):2536–43. Available at: http://www.nejm.org/doi/full/10.1056/NEJMoa0805715. 10.1056/NEJMoa0805715 19516034

[16] Kucharski AJ, Funk S, Eggo RM, Mallet H-P, Edmunds WJ, Nilles EJ. Transmission Dynamics of Zika Virus in Island Populations: A Modelling Analysis of the 2013-14 French Polynesia Outbreak. PLoS Negl Trop Dis [Internet]. 2016 May;10(5):e0004726. Available at: http://ovidsp.ovid.com/ovidweb.cgi?T=JS&CSC=Y&NEWS=N&PAGE=fulltext&D=emexa&AN=610558104. 10.1371/journal.pntd.0004726 PMC4871342 27186984

[17] Coelho AVC, Crovella S. Microcephaly Prevalence in Infants Born to Zika Virus-Infected Women: A Systematic Review and Meta-Analysis. Int J Mol Sci [Internet]. 2017 5 [cited 2017 Oct 4];18(8):1714. Available at: https://www.ncbi.nlm.nih.gov/pmc/articles/PMC5578104/. 10.3390/ijms18081714 PMC5578104 28783051

[18] WHO. Zika causality statement [Internet]. Emergencies. Geneva; 2016 Sep [cited 2018 Jul 11]. Available at: http://www.who.int/emergencies/zika-virus/causality/en/.

[19] Johansson MA (2016). Zika and the risk of microcephaly (vol 375, pg 1, 2016). N Engl J Med.

[20] Krauer F, Riesen M, Reveiz L, Oladapo OT, Martínez-Vega R, Porgo T V., et al. Zika Virus Infection as a Cause of Congenital Brain Abnormalities and Guillain-Barré Syndrome: Systematic Review. von Seidlein L, editor. PLOS Med [Internet]. 2017 3 [cited 2017 Sep 18];14(1):e1002203. Available at: http://dx.plos.org/10.1371/journal.pmed.1002203.. 10.1371/journal.pmed.1002203 PMC5207634 28045901

[21] O’Malley PA (2016). Zika virus: what we know and do not know. Clin Nurse Spec [Internet].

[22] Rasmussen SA, Meaney-Delman DM, Petersen LR, Jamieson DJ. Studying the Effects of Emerging Infections on the Fetus: Experience with West Nile and Zika Viruses. Birth Defects Res. [Internet]. 2017 15;109(5):363–71. Available at: http://ovidsp.ovid.com/ovidweb.cgi?T=JS&CSC=Y&NEWS=N&PAGE=fulltext&D=emexb&AN=619485739. 10.1002/bdr2.1006 PMC7161891 28398684

[23] Campos MC, Dombrowski JG, Phelan J, Marinho CRF, Hibberd M, Clark TG, et al. Zika might not be acting alone: Using an ecological study approach to investigate potential co-acting risk factors for an unusual pattern of microcephaly in Brazil. Roques P, editor. PLoS One [Internet]. 2018 15 [cited 2019 Jan 7];13(8):e0201452. Available at: http://dx.plos.org/10.1371/journal.pone.0201452. 10.1371/journal.pone.0201452 PMC6093667 30110370

[24] Gallo LG, McKeown S, Araújo WN, Velez MP. Risk factors for microcephaly associated with Zika virus infection during pregnancy: a systematic review and meta-analysis [Internet]. PROSPERO 2018 CRD42018088075. 2018 Feb [cited 2019 Jan 8]. Available at: http://www.crd.york.ac.uk/PROSPERO/display_record.php?ID=CRD42018088075.

[25] Moher D, Liberati A, Tetzlaff J, Altman DG, Group TP. Preferred Reporting Items for Systematic Reviews and Meta-Analyses: The PRISMA Statement. PLoS Med [Internet]. 2009 21 [cited 2019 Jan 8];6(7):e1000097. Available at: https://dx.plos.org/10.1371/journal.pmed.1000097.

[26] Aragão MFVV, Holanda AC, Brainer-Lima AM, Petribu NCL, Castillo M, van der Linden V (2017). Nonmicrocephalic infants with congenital Zika syndrome suspected only after neuroimaging evaluation compared with those with microcephaly at birth and postnatally: how large is the Zika virus “iceberg”?. Am J Neuroradiol [Internet].

[27] Schaub B, Gueneret M, Jolivet E, Decatrelle V, Yazza S, Gueye H, et al. Ultrasound imaging for identification of cerebral damage in congenital Zika virus syndrome: a case series. Lancet Child Adolesc Heal [Internet]. 2017 1 [cited 2019 Apr 10];1(1):45–55. Available at: http://ovidsp.ovid.com/ovidweb.cgi?T=JS&CSC=Y&NEWS=N&PAGE=fulltext&D=emexb&AN=618840029. 10.1016/S2352-4642(17)30001-9 30169227

[28] Krow-Lucal ER, de Andrade MR, Cananéa JNA, Moore CA, Leite PL, Biggerstaff BJ, et al. Association and birth prevalence of microcephaly attributable to Zika virus infection among infants in Paraíba, Brazil, in 2015-16: a case-control study. Lancet Child Adolesc Heal [Internet]. 2018 [cited 2019 Apr 10];2(3):205–13. Available at: http://www.ncbi.nlm.nih.gov/pubmed/30169255. 10.1016/S2352-4642(18)30020-8 30169255

[29] Honein MA, Dawson AL, Petersen EE, Jones AM, Lee EH, Yazdy MM (2017). Birth defects among fetuses and infants of US women with evidence of possible Zika virus Infection during Pregnancy. JAMA [Internet].

[30] Kumar M, Ching L, Astern J, Lim E, Stokes AJ, Melish M, et al. Prevalence of Antibodies to Zika Virus in Mothers from Hawaii Who Delivered Babies with and without Microcephaly between 2009–2012. PLoS Negl Trop Dis [Internet]. 2016 Dec;10:e0005262. Available at: http://ovidsp.ovid.com/ovidweb.cgi?T=JS&CSC=Y&NEWS=N&PAGE=fulltext&D=emexa&AN=613987271. 10.1371/journal.pntd.0005262 PMC5215948 27997547

[31] Brasil P, Pereira JP, Moreira ME, Nogueira RMR, Damasceno L, Wakimoto M (2016). Zika virus infection in pregnant women in Rio de Janeiro. N Engl J Med [Internet].

[32] Pomar L, Malinger G, Benoist G, Carles G, Ville Y, Rousset D (2017). Association between Zika virus and fetopathy: a prospective cohort study in French Guiana. Ultrasound Obstet Gynecol [Internet].

[33] Sanz Cortes M, Rivera AM, Yepez M, Guimaraes C V, Diaz Yunes I, Zarutskie A, et al. Clinical Assessment and Brain Findings in a Cohort of Mothers, Fetuses and Infants Infected with Zika Virus. Am J Obstet Gynecol [Internet]. 2018; Available at: http://ovidsp.ovid.com/ovidweb.cgi?T=JS&CSC=Y&NEWS=N&PAGE=fulltext&D=medp&AN=29353032. 10.1016/j.ajog.2018.01.012 29353032

[34] Shiu C, Starker R, Kwal J, Bartlett M, Crane A, Greissman S (2018). Zika virus testing and outcomes during pregnancy, Florida, USA, 2016. Emerg Infect Dis [Internet].

[35] Vargas A, Saad E, Dimech GS, Santos RH, Sivini MAVC, Albuquerque LC (2016). Characteristics of the first cases of microcephaly possibly related to Zika virus reported in the metropolitan region of Recife, Pernambuco state, Brazil. Epidemiol E Serv Saude [Internet].

[36] Franca GVA, Schuler-Faccini L, Oliveira WK de W De, Henriques CMP, Carmo EH, Pedi VD, et al. Congenital Zika virus syndrome in Brazil: a case series of the first 1501 livebirths with complete investigation. Lancet [Internet]. 2016 27 [cited 2016 Sep 3];388(10047):891–7. Available at: http://proxy.queensu.ca/login?url=http://search.ebscohost.com/login.aspx?direct=true&db=cin20&AN=117776094&site=ehost-live. 10.1016/S0140-6736(16)30902-3 27372398

[37] Ventura LO, Ventura C V, Lawrence L, van der Linden V, van der Linden A, Gois AL, et al. Visual impairment in children with congenital Zika syndrome. J Aapos [Internet]. 2017 Aug;21(4):295–9. Available at: http://ovidsp.ovid.com/ovidweb.cgi?T=JS&CSC=Y&NEWS=N&PAGE=fulltext&D=emexa&AN=616795318. 10.1016/j.jaapos.2017.04.003 28450178

[38] Wells GA, Shea B, O’Connell D, Peterson J, Welch V, Losos M, et al. The Newcastle-Ottawa Scale (NOS) for assessing the quality of nonrandomised studies in meta-analyses [Internet]. Ottawa Hospital Research Institute. 2000 [cited 2019 Jan 4]. Available at: http://www.ohri.ca/programs/clinical_epidemiology/oxford.asp.

[39] Modesti PA, Reboldi G, Cappuccio FP, Agyemang C, Remuzzi G, Rapi S, et al. Panethnic Differences in Blood Pressure in Europe: A Systematic Review and Meta-Analysis. Fuchs FD, editor. PLoS One [Internet]. 2016 25 [cited 2019 Apr 10];11(1):e0147601. Available at: https://dx.plos.org/10.1371/journal.pone.0147601. 10.1371/journal.pone.0147601 PMC4725677 26808317

[40] WHO. Avaliação de bebês com microcefalia no contexto do vírus Zika - Orientações provisórias [Internet]. Geneva; 2016 [cited 2016 Aug 23]. (3). Report No.: 16. Available at: http://apps.who.int/iris/bitstream/10665/204475/8/WHO_ZIKV_MOC_16.3_por.pdf?ua=1.

[41] Matos SB de, Meyer R, Lima FW De M. Citomegalovírus: uma Revisão da Patogenia, Epidemiologia e Diagnostico da Infecção. Rev Saúde Com [Internet]. 2011 [cited 2016 Aug 27];7(1):55–7. Available at: http://www.uesb.br/revista/rsc/v7/v7n1a05.pdf.

[42] Baldotto SB, Oliveira PP, Antunes RM, Oliveira PD de, Feitosa PP, Pereira DA. Toxoplasmose com Repercussão Neurológica: Relato de caso. 2015 [cited 2016 Aug 27];9(28):34. Available at: http://fait.revista.inf.br/imagens_arquivos/arquivos_destaque/zGYpCP1yStA225t_2015-9-28-10-50-31.pdf.

[43] Mitsuka-Breganó R, Lopes-Mori FMR, Navarro IT. Toxoplasmose adquirida na gestação e congênita: vigilância em saúde, diagnóstico, tratamento e condutas. EDUEL; 2010.

[44] Kimberlin D. Neonatal herpes simplex infection. Clin Microbiol Rev [Internet]. 2004 [cited 2016 Aug 27]; Available at: http://cmr.asm.org/content/17/1/1.short. 10.1128/CMR.17.1.1-13.2004 PMC321459 14726453

[45] Patrick M, Ken R, Michael P. Medical Microbiology. Vol. 7, Saunders. 2014. 1023 p.

[46] Paixão ES, Barreto F, Teixeira M da GG, Costa M da CNCN, Rodrigues LC. History, Epidemiology, and Clinical Manifestations of Zika: A Systematic Review. Am J Public Health [Internet]. 2016 Apr;106(4):606–12. Available at: http://proxy.queensu.ca/login?url=http://search.ebscohost.com/login.aspx?direct=true&db=cin20&AN=113642877&site=ehost-live. 10.2105/AJPH.2016.303112 PMC4816002 26959260

[47] Saad T, Pennae-Costa AA, de Goes FV, de Freitas M, de Almeida JV, de Santa Ignez LJ (2018). Neurological manifestations of congenital Zika virus infection. Childs Nerv Syst [Internet].

[48] da Silva Pone MV, Pone SM, Zin AA, Barros Mendes PH, Aibe MS, de Aguiar EB (2018). Zika virus infection in children: epidemiology and clinical manifestations. Childs Nerv Syst [Internet]..

[49] Soriano-Arandes A, Rivero-Calle I, Nastouli E, Espiau M, Frick MA, Alarcon A (2018). What we know and what we don’t know about perinatal Zika virus infection: a systematic review. Expert Rev Antiinfective Ther [Internet].

[50] Al-Qaraghouli M, Fang YMV. Effect of Fetal Sex on Maternal and Obstetric Outcomes. Front Pediatr [Internet]. 2017 19;5:144. Available at: http://www.ncbi.nlm.nih.gov/pubmed/28674684. 10.3389/fped.2017.00144 PMC5476168 28674684

[51] Guerra-Silveira F, Abad-Franch F (2013). Sex Bias in infectious disease epidemiology: patterns and processes. PLoS One.

[52] Verburg PE, Tucker G, Scheil W, Erwich JJHM, Dekker GA, Roberts CT. Sexual Dimorphism in Adverse Pregnancy Outcomes - A Retrospective Australian Population Study 1981-2011. PLoS One [Internet]. 2016 [cited 2019 Sep 30];11(7):e0158807. Available at: http://www.ncbi.nlm.nih.gov/pubmed/27398996. 10.1371/journal.pone.0158807 PMC4939964 27398996

[53] Kanmaz AG, İnan AH, Beyan E, Karataşlı V, Çakır İ, Budak A, et al. Effects of fetal gender and low first trimester aneuploidy screening markers on preterm birth. J Gynecol Obstet Hum Reprod [Internet]. 2019; Available at: https://linkinghub.elsevier.com/retrieve/pii/S2468784718305129. 10.1016/j.jogoh.2019.01.011 30685427

[54] Tosun G, İnan AH, Kanmaz AG, Biler A, İleri A, Beyan E, et al. Does fetal sex affect placental delivery times? A prospective observational study. J Matern Neonatal Med [Internet]. 2018 16;1–5. Available at: https://www.tandfonline.com/doi/full/10.1080/14767058.2018.1488163. 10.1080/14767058.2018.1488163 29886800

[55] Mondal D, Galloway TS, Bailey TC, Mathews F. Elevated risk of stillbirth in males: systematic review and meta-analysis of more than 30 million births. BMC Med [Internet]. 2014 27;12(1):220. Available at: http://bmcmedicine.biomedcentral.com/articles/10.1186/s12916-014-0220-4. 10.1186/s12916-014-0220-4 PMC4245790 25428603

[56] Bernin H, Lotter H. Sex Bias in the Outcome of Human Tropical Infectious Diseases: Influence of Steroid Hormones. J Infect Dis [Internet]. 2014 15 [cited 2019 Sep 30];209(suppl 3):S107–13. Available at: https://academic.oup.com/jid/article-lookup/doi/10.1093/infdis/jit610. 10.1093/infdis/jit610 24966190

[57] Rawal G, Yadav S, Kumar R (2016). Zika virus: an overview. J Fam Med Prim Care [Internet].

[58] Mitchell PK, Mier-y-Teran-Romero L, Biggerstaff BJ, Delorey MJ, Aubry M, Cao-Lormeau V-M, et al. Reassessing Serosurvey-Based Estimates of the Symptomatic Proportion of Zika Virus Infections. Am J Epidemiol [Internet]. 2019 1 [cited 2019 Apr 7];188(1):206–13. Available at: https://academic.oup.com/aje/article/188/1/206/5085261. 10.1093/aje/kwy189 PMC6321808 30165474

[59] Hussain A, Ali F, Latiwesh OB, Hussain S. A Comprehensive Review of the Manifestations and Pathogenesis of Zika Virus in Neonates and Adults. Cureus [Internet]. 2018 12;10(9):e3290. Available at: http://www.ncbi.nlm.nih.gov/pubmed/30443460. 10.7759/cureus.3290 PMC6235632 30443460

[60] Shapiro-Mendoza CK, Rice ME, Galang RR, Fulton AC, VanMaldeghem K, Prado MV, et al. Pregnancy Outcomes After Maternal Zika Virus Infection During Pregnancy – U.S. Territories, January 1, 2016-April 25, 2017. MMWR Morb Mortal Wkly Rep [Internet]. 2017 16;66(23):615–21. Available at: http://www.cdc.gov/mmwr/volumes/66/wr/mm6623e1.htm. 10.15585/mmwr.mm6623e1 PMC5657842 28617773

[61] Haby MM, Pinart M, Elias V, Reveiz L (2018). Prevalence of asymptomatic Zika virus infection: a systematic review. Bull World Health Organ [Internet].

[62] Yamada H, Tanimura K, Tairaku S, Morioka I, Deguchi M, Morizane M (2018). Clinical factor associated with congenital cytomegalovirus infection in pregnant women with non-primary infection. J Infect Chemother [Internet].

[63] Peters R, Stevenson M. Zika virus diagnosis: challenges and solutions. Clin Microbiol Infect [Internet]. 2019 Feb [cited 2019 Mar 26];25(2):142–6. Available at: https://linkinghub.elsevier.com/retrieve/pii/S1198743X18307742. 10.1016/j.cmi.2018.12.002 30553031

[64] Pawluk MS, Campanã H, Gili JA, Comas B, Giménez LG, Villalba MJ (2014). Determinantes sociales adversos y riesgo Para anomalías congénitas seleccionadas. Arch Argent Pediatr.

[65] Puthussery S. Perinatal outcomes among migrant mothers in the United Kingdom: Is it a matter of biology, behaviour, policy, social determinants or access to health care? Best Pract Res Clin Obstet Gynaecol [Internet]. 2016;32:39–49. Available at: http://dx.doi.org/10.1016/j.bpobgyn.2015.09.003. 10.1016/j.bpobgyn.2015.09.003 26527304

[66] Amjad S, MacDonald I, Chambers T, Osornio-Vargas A, Chandra S, Voaklander D (2019). Social determinants of health and adverse maternal and birth outcomes in adolescent pregnancies: A systematic review and meta-analysis. Paediatr Perinat Epidemiol.

[67] de Araújo TVB, de XRA, DDB M-F, Souza WV, Monntarroyos UR, de MAP (2018). Association between microcephaly, Zika virus infection , and other risk factors in Brazil: final report of a case-control study. Lancet Infect Dis [Internet].

[68] Frank C, Faber M, Stark K (2016). Causal or not: applying the Bradford Hill aspects of evidence to the association between Zika virus and microcephaly. EMBO Mol Med.

[69] M.G. A, Schwartz DA, Alvarado MG, Schwartz DA. Zika Virus Infection in Pregnancy, Microcephaly, and Maternal and Fetal Health: What We Think, What We Know, and What We Think We Know. Arch Pathol Lab Med [Internet]. 2017;141(1):26–32. Available at: http://proxy.queensu.ca/login?url=http://search.ebscohost.com/login.aspx?direct=true&db=cin20&AN=120544273&site=ehost-live. 10.5858/arpa.2016-0382-RA 27636525

[70] Williamson J. Establishing the teratogenicity of Zika and evaluating causal criteria. Synthese [Internet]. 2018;1–14. Available at: https://doi.org/10.1007/s11229-018-1866-9.

[71] Zhu Z, Chan JF-W, Tee K-M, Choi GK-Y, Lau SK-P, Woo PC-Y (2016). Comparative genomic analysis of pre-epidemic and epidemic Zika virus strains for virological factors potentially associated with the rapidly expanding epidemic. Emerg Microbes Infect [Internet].

[72] Metsky HC, Matranga CB, Wohl S, Schaffner SF, Freije CA, Winnicki SM, et al. Zika virus evolution and spread in the Americas. Nature [Internet] 2017;546(7658):411–415. Available at: http://dx.doi.org/10.1038/nature22402. 10.1038/nature22402 PMC5563848 28538734

