# Supplementary material for: Another piece of the Zika puzzle: assessing the associated factors to microcephaly in a systematic review and meta-analysis
Source: BMC Public Health. 2020 Jun 1;20:827. doi: 10.1186/s12889-020-08946-5 (PMC7266116; doi:10.1186/s12889-020-08946-5)
Supplement: Supplementary file 2 — Additional file 2 Additional Table 2. Standardized table sent to all the corresponding authors. [file 12889_2020_8946_MOESM2_ESM.docx]

| (Only confirmed or probable Zika virus cases) | Microcephaly + (Head circunference <= -2SD for age and sex) | Microcephaly - |
| --- | --- | --- |
| Population (N) |  |  |
| Mother's age (Mean and SD) |  |  |
| Maternal ethnicity (proportion of non-white) |  |  |
| Maternal studies' years (Mean and SD) |  |  |
| Sex (proportion of boys ) |  |  |
| Gestational age at birth (Mean and SD) |  |  |
| Presence of symptoms during gestation (N) |  |  |
| Smoking habits and/or use of alcohol and/or other drugs during pregnancy (N) |  |  |
| Presence of materbal comorbidities during pregnancy (N) |  |  |
| Infected in the first gestational trimester (N) |  |  |
| Infected in the second gestational trimester (N) |  |  |
| Infected in the third gestational trimester (N) |  |  |
| vaccinated - Yellow fever vaccine (N) |  |  |
| vaccinated - Other vaccines (N) |  |  |
