# Supplementary material for: Another piece of the Zika puzzle: assessing the associated factors to microcephaly in a systematic review and meta-analysis
Source: BMC Public Health. 2020 Jun 1;20:827. doi: 10.1186/s12889-020-08946-5 (PMC7266116; doi:10.1186/s12889-020-08946-5)
Supplement: Supplementary file 3 — Additional file 3 Additional Table 3. Newcastle-Ottawa Quality Assessment Scale - retrospective studies. [file 12889_2020_8946_MOESM3_ESM.docx]

| Quality assessment criteria | Aragao et al 2017 | Halai et al  2017 | Krow-Lucal et al  2017 | Kumar, 2016 |
| --- | --- | --- | --- | --- |
| Is the case definition adequate? | * | * | * | * |
| Representativeness of the cases | * | * | - | * |
| Selection of Controls | - | * | * | * |
| Definition of Controls | * | * | * | * |
| Comparability of cases and controls on the basis of the design or analysis | * | * | * | * |
| Ascertainment of exposure | - | * | - | * |
| Same method of ascertainment for cases and controls | * | * | * | * |
| Non-Response rate | - | - | - | * |
|  | 5 | 7 | 5 | 8 |
