# Supplementary material for: Another piece of the Zika puzzle: assessing the associated factors to microcephaly in a systematic review and meta-analysis
Source: BMC Public Health. 2020 Jun 1;20:827. doi: 10.1186/s12889-020-08946-5 (PMC7266116; doi:10.1186/s12889-020-08946-5)
Supplement: Supplementary file 4 — Additional file 4 Additional Table 4. Newcastle-Ottawa Quality Assessment Scale - cohort studies. [file 12889_2020_8946_MOESM4_ESM.docx]

| Quality assessment criteria | **Pomar *et al.*, 2017** | **Honein *et al.*, 2017** | **Cortes *et al.*, 2017** | **Shiu *et al.*, 2016** | **Brasil *et al.*, 2016** |
| --- | --- | --- | --- | --- | --- |
| **Selection** | | | | | |
| Representativeness of the exposed cohort | * | * | * | * | * |
| Selection of the non exposed cohort | * | * | * | * | * |
| Ascertainment of exposure? | * | * | * | * | * |
| Demonstration that outcome of interest was not present at start of study? | - | * | - | - | * |
| **Comparability** | | | | | |
| Comparability of cohorts on the basis of the design or analysis | * | * | * | * | ** |
| **Outcome** | | | | | |
| Assessment of outcome | * | * | * | * | - |
| Was follow-up long enough for outcomes to occur | * | * | * | * | * |
| Adequacy of follow up of cohorts | - | - | - | - | _ |
|  | 6 | 7 | 6 | 6 | 7 |
