# Supplementary material for: Another piece of the Zika puzzle: assessing the associated factors to microcephaly in a systematic review and meta-analysis
Source: BMC Public Health. 2020 Jun 1;20:827. doi: 10.1186/s12889-020-08946-5 (PMC7266116; doi:10.1186/s12889-020-08946-5)
Supplement: Supplementary file 5 — Additional file 5 Additional Table 5. Newcastle-Ottawa Assessment Scale adapted for cross-sectional studies. [file 12889_2020_8946_MOESM5_ESM.docx]

| Quality assessment criteria | **Ventura, *et al.*, 2017** |
| --- | --- |
| **Selection** | |
| Representativeness of the sample | - |
| Sample size | - |
| Non-respondents | * |
| Ascertainment of the exposure (risk factor) | ** |
| **Comparability** | |
| Comparability of subjects in different outcome groups on the basis of design or analysis. Confounding factors controlled | ** |
| **Outcome** | |
| Assessment of outcome | ** |
| Statistical test | * |
|  | 8 |
