# Supplementary material for: Another piece of the Zika puzzle: assessing the associated factors to microcephaly in a systematic review and meta-analysis
Source: BMC Public Health. 2020 Jun 1;20:827. doi: 10.1186/s12889-020-08946-5 (PMC7266116; doi:10.1186/s12889-020-08946-5)
Supplement: Supplementary file 6 — Additional file 6 Additional Table 6. Newcastle-Ottawa Assessment Scale adapted for case reports and case series . [file 12889_2020_8946_MOESM6_ESM.docx]

| Domains Leading explanatory questions^a^ | **Schaub *et al*., 2017** | **Vargas *et al*., 2016** | **França *et al*., 2016** |
| --- | --- | --- | --- |
| **Selection** | | | |
| 1. Does the patient(s) represent(s) the whole experience of the investigator (centre) or is the selection method unclear to the extent that other patients with similar presentation may not have been reported? | ***** | ***** | ***** |
| **Ascertainment** | | | |
| 1. Was the exposure adequately ascertained? | ***** | ***** | **-** |
| 1. Was the outcome adequately ascertained? | ***** | ***** | ***** |
| **Causality** | | | |
| 1. Were other alternative causes that may explain the observation ruled out? ^+^ | ***** | ***** | **-** |
| 1. Was there a challenge/rechallenge phenomenon? ^+^ | **DNI^!^** | **DNI^!^** | **DNI^!^** |
| 1. Was there a dose–response effect? ^+^ | **-** | **-** | **-** |
| 1. Was follow-up long enough for outcomes to occur? | ***** | **-** | **-** |
| **Reporting** | | | |
| 1. Is the case(s) described with sufficient details to allow other investigators to replicate the research or to allow practitioners make inferences related to their own practice? | ***** | ***** | ***** |
|  | **6** | **5** | **3** |

^a^ Adapted from the Newcastle-Ottawa Quality Assessment Scale. Source: Murad MH, Sultan S, Haffar S, Bazerbachi F, Mohammad D, Murad H. Methodological quality and synthesis of case series and case reports. BMJ evidence-based Med [Internet]. 2018 [cited 2019 Apr 2]; 23(2):60–3. Available at: http://www.ncbi.nlm.nih.gov/pubmed/29420178

^+^ Questions 4, 5 and 6 are mostly relevant to cases of adverse drug events.

^!^ Does not apply.
