# Supplementary figures and images for: Another piece of the Zika puzzle: assessing the associated factors to microcephaly in a systematic review and meta-analysis
Source: BMC Public Health. 2020 Jun 1;20:827. doi: 10.1186/s12889-020-08946-5 (PMC7266116; doi:10.1186/s12889-020-08946-5)

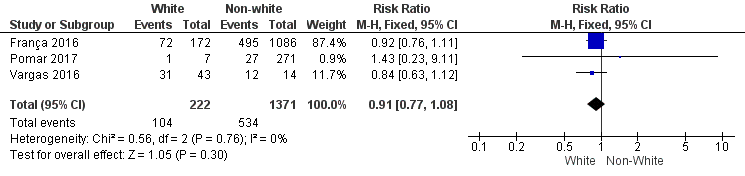


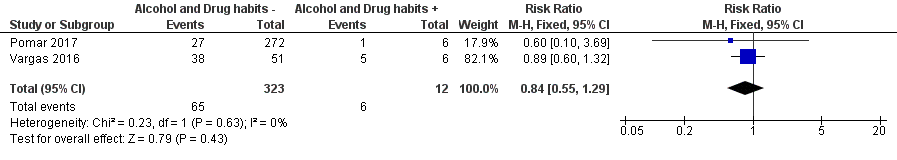


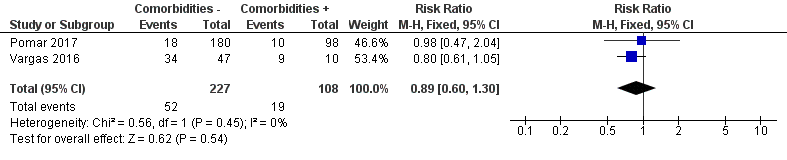


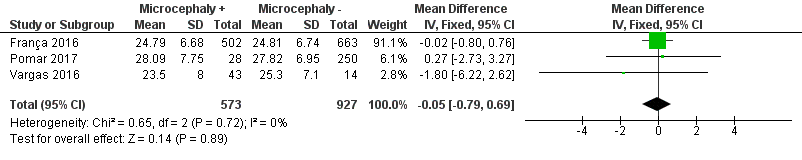


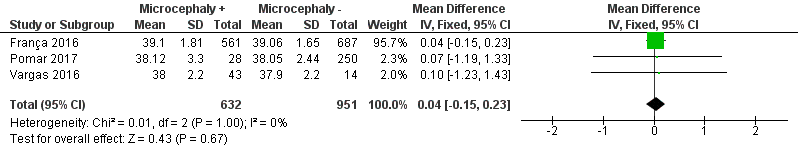

Supplement: Supplementary file 7 — Additional file 7 Additional Fig. 01. Meta-analysis forest plot of prospective studies. Additional Fig. 01a. Maternal ethnicity. Additional Fig. 01b. Smoking habits and/or consumption of alcohol and/or other substances during pregnancy. Additional Fig. 01c. Maternal comorbidities during pregnancy. Additional Fig. 01d. Mean maternal age. Additional Fig. 01e. Mean gestational age at the birth. [file 12889_2020_8946_MOESM7_ESM.docx]

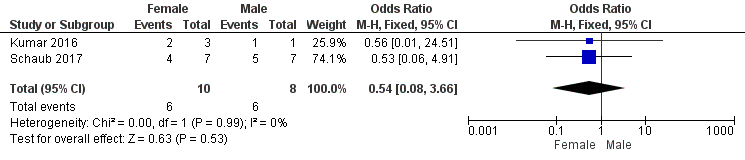


**
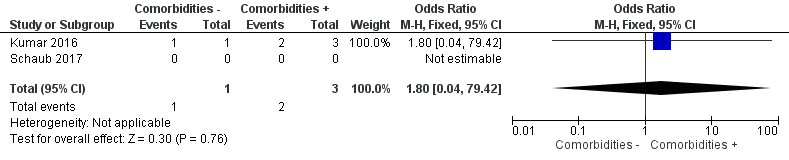
**

Supplement: Supplementary file 8 — Additional file 8 Additional Fig. 02. Meta-analysis forest plot of retrospective studies. Additional Fig. 02a. Sex of newborns/fetuses in case-control studies. Additional Fig. 02b. Maternal comorbidities during pregnancy. [file 12889_2020_8946_MOESM8_ESM.docx]
